# Stimuli-Responsive Block Copolymer-Based Assemblies for Cargo Delivery and Theranostic Applications

**DOI:** 10.3390/polym8070268

**Published:** 2016-07-22

**Authors:** Jun Yin, Yu Chen, Zhi-Huang Zhang, Xin Han

**Affiliations:** Department of Polymer Science and Engineering, School of Chemistry and Chemical Engineering, Hefei University of Technology and Anhui Key Laboratory of Advanced Functional Materials and Devices, Hefei 230009, China; chenyu0818hfut@163.com (Y.C.); zhangzhihuang0930@163.com (Z.-H.Z.); 13003090819@163.com (X.H.)

**Keywords:** block copolymers, stimuli-responsive, nanocarriers, drug delivery, theranostic

## Abstract

Although a number of tactics towards the fabrication and biomedical exploration of stimuli-responsive polymeric assemblies being responsive and adaptive to various factors have appeared, the controlled preparation of assemblies with well-defined physicochemical properties and tailor-made functions are still challenges. These responsive polymeric assemblies, which are triggered by stimuli, always exhibited reversible or irreversible changes in chemical structures and physical properties. However, simple drug/polymer nanocomplexes cannot deliver or release drugs into the diseased sites and cells on-demand due to the inevitable biological barriers. Hence, utilizing therapeutic or imaging agents-loaded stimuli-responsive block copolymer assemblies that are responsive to tumor internal microenvironments (pH, redox, enzyme, and temperature, etc.) or external stimuli (light and electromagnetic field, etc.) have emerged to be an important solution to improve therapeutic efficacy and imaging sensitivity through rationally designing as well as self-assembling approaches. In this review, we summarize a portion of recent progress in tumor and intracellular microenvironment responsive block copolymer assemblies and their applications in anticancer drug delivery and triggered release and enhanced imaging sensitivity. The outlook on future developments is also discussed. We hope that this review can stimulate more revolutionary ideas and novel concepts and meet the significant interest to diverse readers.

## 1. Introduction

With the increasing numbers of cancerous types and patients, cancer diagnosis and therapy have received a growing attention of chemists and biologists. A range of treatment protocols has been developed, including surgery, radiotherapy, and chemotherapy. However, conventional chemotherapy mainly relies on small molecule anticancer drugs, leading to indiscriminate killing of both cancerous and healthy tissues/cells [1,2]. In addition, chemotherapy with the aid of small molecule drugs is suffered from many obstacles, such as low solubility, poor pharmacokinetics, undesirable biodistribution, inefficient cellular uptake, and an inability to target desired locations [3,4]. Similarly, the new kind of biological drugs such as proteins, antibodies, and nucleic acids, may be deactivated or degraded during blood circulation before they get to the target sites [5,6]. These undesired side effects largely decreased the therapeutic efficacy and outcome, and the possible multidrug resistance to certain types of cancer cells might further decrease the therapeutic efficacy more or less. Moreover, both systemic and cellular barriers will further hinder the efficient delivery of conventional small molecule drugs to target sites.

To overcome these limitations, polymeric materials based multifunctional delivery systems capable of targeting transport and follow-up site-specific controlled drug release have been developed [7,8,9,10,11,12]. Polymeric materials, especially amphiphilic block copolymers (ABCs) and double hydrophilic block copolymers (DHBCs), have been widely utilized in nanomedicine applications, such as imaging/contrast agent carriers, drug delivery systems (DDS), and integrated theranostic platform owing to their improved water dispersibility, biocompatibility, tunable compositions, extended blood circulation duration, and facile functionalization [13,14,15,16]. Among them, nanoassemblies, including micelles, vesicles, and other complex topological structures, self-assembled from ABCs or DHBCs in water have often been employed [17,18]. They possess excellent stability due to the low critical micellization concentration (CMC) values and the assistance of possible covalent or non-covalent immobilization on the assembly structures. Such assemblies have been broadly applied to encapsulate therapeutic or imaging agents as nanocarriers due to the enhanced solubility, increased drug efficacy, and reduced drug toxicity and degradation compared with small molecule drugs alone.

Nanocarriers can be able to accumulate at tumor sites either through passive targeting by the enhanced permeability and retention (EPR) effect [19,20] or through active targeting by the introduction of targeting moieties that specific for receptors or ligands, enabling precisely control on the drug location, dose, and curative time. Compared with the EPR effect in passive targeting strategy, active targeting has become a new direction of modern nanocarrier design and offers extra advantages of medical demand. The relevant relationship between various polymer structures and correlated functions relies on the assistance of molecular design, subsequent chemical modification, and implementation approaches. Nanocarriers for active targeting need to be modified with affinity ligands, such as functional organic molecules, carbohydrates, peptides, antibodies, and aptamers, which can selectively recognize and bind to specific tissues, cells, or organelles with complementary receptors or antigens [21,22]. It is very important for polymer based delivery systems that the intracellular uptake efficiency can be largely promoted via specific ligand-receptor interactions, resulting in subsequent high-efficiency working within the intracellular microenvironment.

Furthermore, the tunable sizes and diverse assembly morphologies are another two key factors for polymeric assemblies that influences blood circulation time and tumor accumulation [23,24]. It has been well established that nanoparticle carriers with diameters smaller than ~200 nm favor tumor accumulation via EPR effect within tumor tissues because they can avoid clearance by phagocytic uptake and hepatic filtration and their superior deep tumor tissue penetration [25,26]. Recently, nanocarriers with sizes smaller than 100 nm are acknowledged to be more favorable for tumor tissue accumulation [27,28]. On the other hand, worm-like micelles with larger aspect ratio can keep the in vivo circulation for a long time, which is about several times longer than spherical counterparts and result in pronounced tumor accumulation and deep penetration [29,30]. To maintain the individual stability and extend the blood circulation time, the susceptible interactions between nanocarriers and blood plasma proteins should be avoided, thus, biocompatible hydrophilic polymer chains should be incorporated into designed block copolymers and corresponding assemblies. As for stabilizing polymers, poly(ethylene glycol) (PEG), poly(2-hydroxypropyl methacrylate) (PHPMA), poly(2-meth-acryloyloxyethyl phosphorylcholine) (PMPC), and poly(carboxybetaine) are typical representatives because of their protein-resistant and the bypassing of recognition and capture by the reticuloendothelial system (RES) or mononuclear phagocyte system (MPS) [31,32].

Up to now, extensive polymer assemblies have been developed for the delivery of small molecule drugs and imaging reagents, not only protected them from degradation or inactivation in vivo, but also used to optimize their doses and working efficacy. Small molecule drugs can either be physically encapsulated into polymeric assemblies or covalently conjugated onto polymer chains with high drug-loading content and minimal adverse effects on normal organs. In the case of ordinary physical encapsulation, these assemblies can be achieved generally through in situ drug loading by inner hydrophobic core during assembly formation, but the accurately controlled drug release within target tissues or specific cells is difficult to carry out. The major problems are the undesirable drug leaking during blood circulation due to the non-covalent linkage and the inefficient assembly accumulation in the targeting disease site. An efficient and implemented program to solve the drug leaking defect is either attaching drug molecules to the main/side chains of biocompatible amphiphilic block copolymers to form “polymer-drug conjugates” [33] or polymerization of prodrug monomers as a building unit to form so-called “polyprodrugs” [34], allowing for precisely control over drug loading and release, which have already opened up an emerging drug carrying and therapeutic method and generated positive results. However, the degradation and erosion rates of polymeric nanocarriers are highly affected by the nature of the polymer itself as well as the linker between polymer and drug in such systems, which are important for tailoring the nanocarriers for various therapeutic purpose.

Aside from the drug protection from wrapped polymers, the introduction of stimuli-responsive polymers, groups, or linkages into nanocarriers will endow additional routes for triggered release of incorporated therapeutic agents into specific tissues by pH, temperature, enzyme concentration, oxidation-reduction surrounding, electromagnetic field, light, or specific bioactive molecules [35,36,37,38]. Compared to normal tissues, tumor sites exhibit several abnormalities, such as weak acidity, unusual temperatures [39,40], hypoxia [41], and high levels of reactive small molecules [42,43], etc. Upon cellular uptake, therapeutic agents-loaded polymeric nanocarriers constructed from block copolymers that responsive to the innate characteristic of pathologic tissues and cells will be subjected to intracellular pH gradients, oxidative-reductive environment, and reactive oxygen species (ROS) gradients within cytosol and different cell organelles. The responsive polymer chains within designed nanocarriers will undergo structural transformation or directly disassemble into smaller pieces once exposure to specific stimuli, which can obviously enhance site-specific drug release and boost therapeutic efficacy by increasing local concentration at the desired site, no matter through passive or active targeted delivery.

Due to the urgent requirement of accurate diagnosis, imaging technology guided theranostic has become a newly presented research hotspot. Several kinds of imaging approaches like fluorescence imaging, magnetic resonance imaging (MRI), computed tomography (CT), and radionuclide imaging have been developed in turn [44,45,46,47]. By encapsulating imaging agents together with drugs within a single polymeric assemblies, researchers can be able to achieve analysis of drug distribution and release at the target site in real time. Nowadays, the emergence of multi-mode imaging guided theranostic protocol has become the hotspot in nanomedicines, which integrated diagnosis and therapy into a single platform, allowing for simultaneous monitoring and treatment on disease tissues, which is a newly emerging and fast growing research field in current medical science and plays a really important role [48]. Numerous kinds of organic/inorganic nano-scaled assemblies for theranostic were developed, such as polymeric nanoparticles, metal nanoparticles, and carbon materials. However, the inorganic or metallic materials have suffered from toxicity, slow excretion kinetics, and other defects, which limited their application in human body. Polymer based nanocarriers can also be employed as delivery vehicles for imaging agents, holding great promise for the fabrication of multifunctional integrated theranostic formulations.

In this article, some critical achievements and rising trend in stimuli-responsive block copolymer assemblies, which are responsive to tumor and intracellular microenvironments and their applications in the area of bioimaging, drug delivery, and therapeutics are summarized. Different from the traditional focus, herein, aside from those frequently appeared factors (temperature, pH, redox, enzyme, and light), the novel chemical structures and tailor-made functions, such as polyprodrug amphiphiles and responsive linker incorporated self-immolative system, are systematically discussed. Moreover, block copolymer nanocarriers responsive to more than one type of stimuli signal for sequential or synergistic microstructural evolution and programmed drug release will also be discussed. Finally, we present an outlook and perspective concerning the potential usage of structural stable hydrophilic helical poly(phenyl isocyanide) (PPI) contained copolymers in this emergent field of bio-relevant nanocarriers. The inherent helical structure of PPI is an excellent mimic for cell-penetrating peptides (CPPs), which performed as an efficient medium to facilitate intracellular delivery of various cargos. The selection of papers was definitely not easy due to the limited page space. We hope this review will be suitable for diverse readers in the field of chemical, biological, and medical science.

## 2. Temperature-Sensitive Assemblies

The abnormal temperature gradients and sensitivity in tumor tissues are critical physiochemical features as compared with that of normal tissues. For example, the local temperature of breast cancer is obviously higher than that of the surrounding tissues [49]. Thus, the utilization of block copolymers containing thermoresponsive building blocks with adjustable phase transition temperatures can be used for the fabrication of temperature sensitive drug delivery systems, allowing for temperature-triggered drug release. Up to now, a variety of strategies based on a thermoresponsive drug delivery systems have been developed [50,51,52,53]. Herein, the thermo-triggered drug release from block copolymer assemblies possessing volume phase transition temperature near body temperature are discussed. 

**Figure 1:**
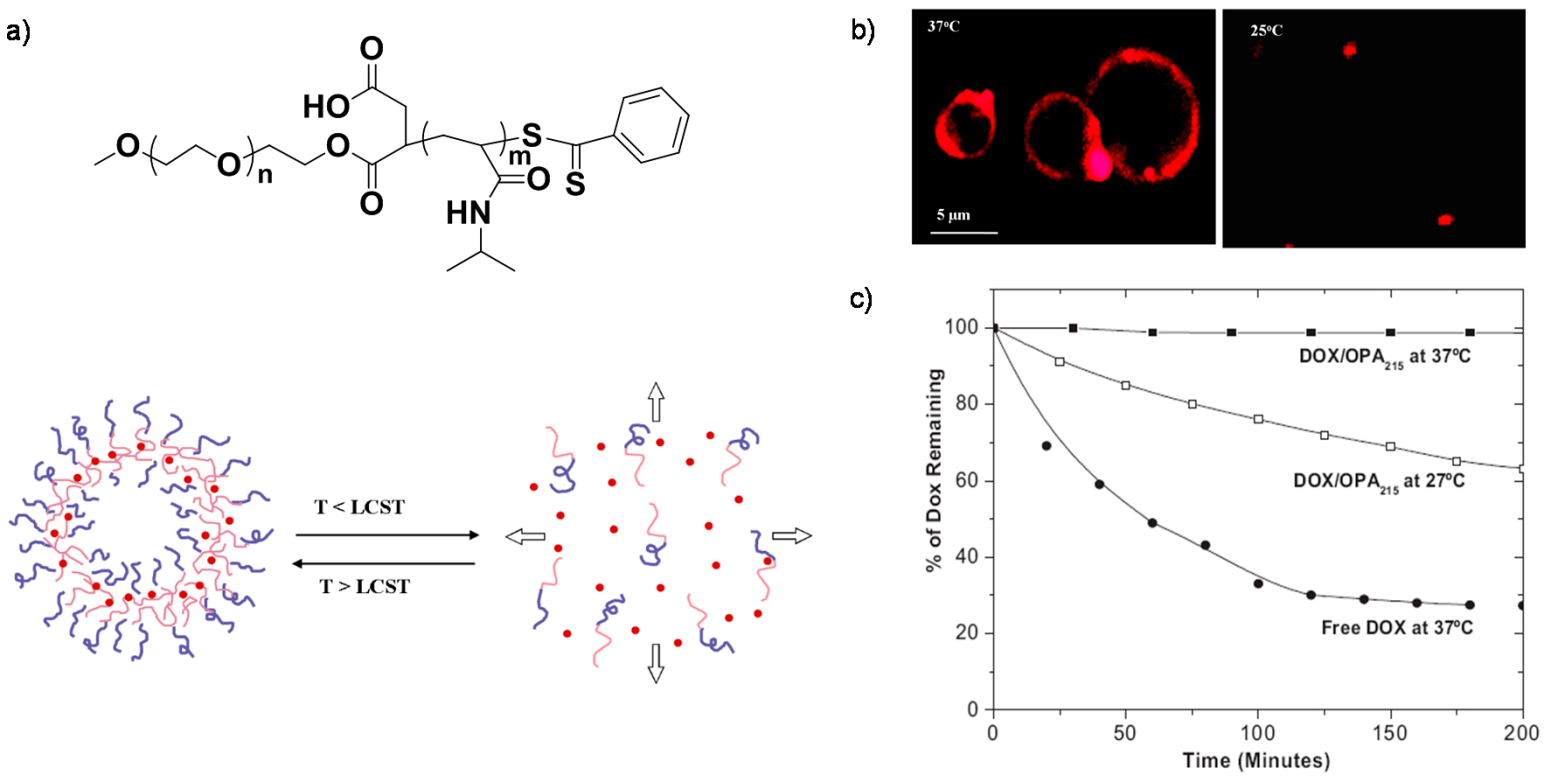
(**a**) Chemical structure of PEO-*b*-PNIPAM copolymers and schematic illustration of thermo-responsive vesicles; (**b**) Fluorescent images of live cells when culturing with PEO-*b*-PNIPAM vesicles at different temperatures, the membrane was labeled with PKH 26; (**c**) Release of Dox from polymer vesicles at 37 and 27 °C. Reproduced with permission from [57].

Representatively, poly(*N*-isopropylacrylamide) (PNIPAM), with a typical lower critical solution temperature (LCST) of ~32 °C [54,55,56], have been widely investigated for drug delivery systems owing to their thermo-responsive and considerable phase transition behaviors. The transition temperatures could be tailored as needed by tuning molecular weights (*M*_W_s) or copolymerization with hydrophobic or hydrophilic monomers to form PNIPAM incorporated block copolymers. For example, Yang and Discher et al. described the synthesis and preparation of thermoresponsive polymersomes from diblock copolymers of PEO-*b*-PNIPAM [57]. The copolymers could become amphiphilic in water once the temperature was increased above body temperature (~37 °C), and self-assemble into polymer vesicles with hydrophobic PNIPAM bilayers, possessing temperature-dependent assembly-disassembly features (Figure 1a). The vesicles could encapsulate both hydrophilic and hydrophobic molecules into their watery interior and hydrophobic bilayers, respectively, and maintain stable structure at body temperature. With a decrease in temperature, vesicle disassembly is triggered with release of the encapsulants, and causing the vesicle fluorescence to disappear (Figure 1b). The local drug release capability endow these vesicles promising for drug delivery (Figure 1c).

Aside from temperature-sensitive DHBCs, a series of other PNIPAM containing thermoresponsive diblock copolymers, including PNIPAM-*b*-poly(styrene) (PNIPAM-PS), PNIPAM-*b*-poly(*n*-butyl methacrylate) (PNIPAM-PBMA), and stearoyl-terminated PNIPAM-C_18_ were reported [58]. All of them can self-assemble into core-shell micelles with PNIPAM as the shell at room temperature. Among them, PNIPAM-PBMA micelles could uptake and release DOX when heated above the LCST of PNIPAM, and showed obvious cytotoxicity. Moreover, biocompatible hydrophobic blocks, such as poly(lactic acid) (PLA) and poly(ε-caprolactone) (PCL), were also used to construct block copolymers together with PNIPAM and corresponding corona-thermoresponsive micelles [59]. For example, Liu et al. reported on the facile synthesis of well-defined amphiphilic and thermoresponsive tadpole-shaped cyclic-linear diblock copolymers, (*c*-PNIPAM)-*b*-PCL, via ring-opening polymerization directly initiating from cyclic precursors [60]. At 20 °C, the (*c*-PNIPAM)-*b*-PCL copolymers self-assembled into spherical micelles consisting of hydrophobic PCL cores and well-solvated coronas of *c*-PNIPAM in aqueous solution. As the Dox-loaded micelle dispersion was placed in a pH 7.4 PBS buffer at 20 °C, ~55% of the loaded Dox was released after 50 h. When the buffer temperature increased to 37 °C (above the LCST of PNIPAM coronas), enhanced release rate of Dox can be clearly observed, and ~84% of the loaded Dox can be released after 50 h. The results confirmed that the rate of drug release from micelles fabricated from PNIPAM contained amphiphilic diblock copolymers can be accelerated at elevated temperatures. For comparison, linear diblock copolymer with comparable molecular weight and composition, (*l*-PNIPAM)-*b*-PCL, was also synthesized. The effect of chain topologies on the thermal phase transition properties and their functions as controlled release drug nanocarriers were systematically investigated. Similar strategy can be also utilized for constructing core-thermoresponsive micelles [61,62].

In addition to PNIPAM, many other thermoresponsive polymers were also developed, including poly(2-isopropyl-2-oxazoline) (PIPO) [63], oligo(ethylene glycol) modified polymers [64], poly(*N*-(2-hydroxypropyl)methacrylamide lactate) (P(HPMA-Lacn)) [65], and elastin-like polymers (ELPs) [66], etc. The phase transition behavior of PIPO is similar to that of PNIPAM. The LCST values of poly(oligo(ethylene glycol) monomethyl ether methacrylate) (POEGMA) are varied with the side length of ethylene glycol units. For example, PMEO_2_MA with two EO units and POEGMA_475_ with nine EO units possess LCST values of ~26 and ~90 °C [64], respectively, and both of them exhibited excellent biocompatibility and low cytotoxicity. Recently, Jiang et al. synthesized a new class of PEG functionalized monodispersed fourth generation dendrimers with generation-dependent thermosensitivity, gemcitabine was then introduced to the periphery of the dendrimers and superior tumor accumulation and penetration capability were declared [67]. The LCST of P(HPMA-Lacn) could be tuned in the range of 10–65 °C by varying the grafting density of lactate-containing side chains [65]. Moreover, ELPs are protein-based materials with the ability to capture drug molecules by undergoing LCST-like phase transition when the polymer solution is heated above its transition temperature [68]. They are widely used in drug delivery systems owing to their excellent biocompatible and biodegradable properties. The assemblies, once formed, are stable either at room or body temperature and showed slowly degradation as well as a sustained drug release. However, it should be noted that conventional polymeric micelles may dissociate into unimers at a concentration lower than the critical micelle concentration (CMC) upon high dilution, such as intravenous injection. Further efforts should consider the stability of such micelles.

## 3. pH-Sensitive Assemblies

**Figure 2:**
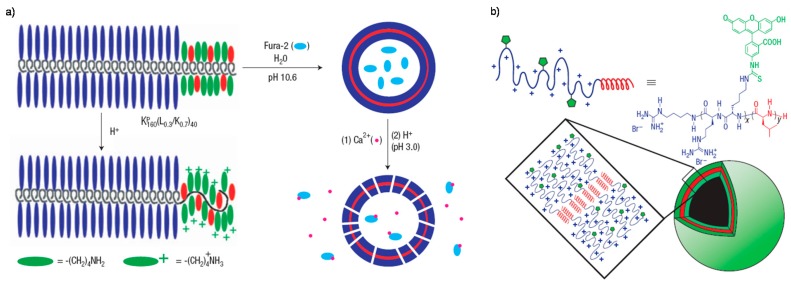
(**a**) Schematic drawing of copolypeptides, its change in conformation with pH, and release of entrapped Fura-2 dye on pH change; (**b**) Schematic diagram of proposed self-assembly of vesicles. Reproduced with permission from [73,74].

Generally, the extracellular pH of most tumor tissues is weakly acidic (pH 6.5–6.8), which is obviously lower than that of normal tissues (pH 7.2–7.4). The pH discrepancy is also usually used as the trigger factor for ultrasensitive pH-responsive delivery of therapeutic and imaging agents. In the past decades, a series of ultrasensitive pH-responsive polymers have been explored [69,70]. Amphiphilic block copolymers consisting of pH-responsive building blocks always exhibit tunable pH-responsive micellization behavior due to the solubility of the amine group containing segments, which can be adjusted via pH-tunable protonation/deprotonation. Typical examples were reported by Bae et al. [71] and Kim et al. [72] that polyhistidine (Phis) containing diblock (PEG-*b*-Phis and PHEMA-*b*-Phis) or triblock (PLA-*b*-PEG-*b*-Phis) copolymers were synthesized and used to construct polymeric assemblies serving as a tumor acidic pH-specific anticancer drug carrier. Under tumor acidic microenvironment, the protonation of PHis blocks might lead to reorganization and deformation of assembly structures, resulting in acidic pH-triggered drug release. Similarly, Bellomo and Wyrsta et al. reported the preparation of amphiphilic diblock copolypeptides containing $K_{X}^{P}$ and $L_{y}$ with varying chain lengths and fractions of hydrophobic residues, where $K^{P}$ = poly(*N*_ε_-2-(2-(2-methoxyethoxy)ethoxy)acetyl-L-lysine and *L* = poly(L-leucine) [73]. These copolypeptides could self-assemble into spherical vesicular assemblies whose size and structure were dictated primarily by the ordered conformations of the polymer segments, in a manner similar to viral capsid assembly. Fluorescent dyes could be encapsulated within the vesicles and showed a pH-dependent release upon lowering pH values (Figure 2a). Moreover, Deming et al. prepared vesicles composed of polyarginine and polyleucine segments, which could entrap water soluble species [74]. The remarkable feature of these vesicles is that the polyarginine segments both direct structure for vesicle formation and provide functionality for efficient intracellular delivery of the vesicles (Figure 2b). The unique synergy between nanoscale self-assembly and inherent peptide functionality provides a new approach for design of multifunctional materials for drug delivery.

Since the early pioneering work reported by Shen and Ryser et al. the concept of pH-triggered hydrolysis of the linkage between polymer scaffold and pendent group in polymeric assemblies was emerged to release specific molecules to targeted areas [75]. Many pH-sensitive polymeric nanocarriers have been developed according to the employment of hydrazine [76,77], acetal [78], ketal [79], and imino [80,81] linkages, which rely on a structure conversion in the mildly acidic environment. These nanocarriers are usually quite stable during blood circulation, but the rate of cargo release from them is pH-dependent with a high rate obtained at low pH values and a low rate was observed at physiological pH. For example, acetal was frequently explored as the acid labile functionalities. Fréchet et al. reported the preparation of linear-dendritic block copolymers comprising PEO and polylysine, then, hydrophobic groups were attached to the dendrimer periphery by acid-cleavable cyclic acetal [82]. These copolymers could form stable micelles in aqueous solution at neutral pH but disintegrate into unimers at mildly acidic pH due to the acetal-aldehyde conversion. This distinctive feature endows acid-responsive linkages to be further utilized in the design of anticancer drug carriers. In some cases, the hydrolysis of the linkage is expected to supplement the limited buffering capacity during endosomal escape and convert charged chains to neutral chains, which reduce their interactions with endogenous charged moieties, e.g., DNA.

The immaculate nanoparticle-based delivery systems should be capable of fitting the demands of a series of stages during delivery procedure, such as prolonged circulation, enhanced accumulation in the tumor, facilitated cellular internalization, and rapid release of the active drug molecules in the tumor cells. However, the optimal strategy for improving one aspect often conflicts with the other, especially the conflict between enhanced cellular uptake and prolonged blood circulation. To get a comprehensive effect, Wang et al. reported a biocompatible and tumor-pH responsive polymeric nanoparticles based on an acid-degradable amide bond (Dlinkm) bridged PEG and poly(D,L-lactide) (PDLLA) block copolymers, denoted as PEG-*Dlinkm*-PDLLA, for improved cancer therapy [83]. The amphiphilic copolymer could self-assemble into PEGylated nanoparticles with a diameter of ~100 nm, which could accumulate preferentially at tumor site through conventional EPR effect. After extravasation into the tumor site, the characteristic tumor acidity (pH 6.5–7.0) triggered the cleavage of the *Dlinkm* linkage, leading to the release of the PEG corona as well as the increase of zeta potential of the nanoparticle, both of which in turn facilitated cellular uptake. With loading of docetaxel (DTXL), the enhanced internalization would also improve in vitro and in vivo therapeutic efficacy. Following the same concept, the same group reported the preparation of *Dlinkm* linked PEG-*Dlinkm*-PLGA block copolymers [84]. After encapsulating siRNA into the nanoparticles through the cationic lipid-assisted double emulsion method, the formed ^dPEG^NP_PLGA/siRNA_ composite nanoparticles could not only prolong circulation in the blood and accumulate preferentially in tumor sites, but also facilitated cellular uptake by detachment of the PEG corona in response to tumor pH and rapid release of siRNA within tumor cells by replacing the PLA block with PLGA copolymer.

To enhance the stability of polymeric micelles, cross-linked micellar structure is an excellent solution. Recently, Hu et al. reported the fabrication of core cross-linked (CCL) polymeric micelles, which covalently labeled with DOTA(Gd) and green-emitting fluorophores within pH-responsive cores [85], serving as a dual-modality magnetic resonance (MR)/fluorescence imaging agents. The acidic pH-triggered turn-on and enhancement of signal intensities for both imaging modalities were declared. Compared with non-cross-linked diblock precursor, CCL micelles demonstrated better MR and fluorescence imaging performance due to structural stability endowed by the CCL protocol.

**Figure 3:**
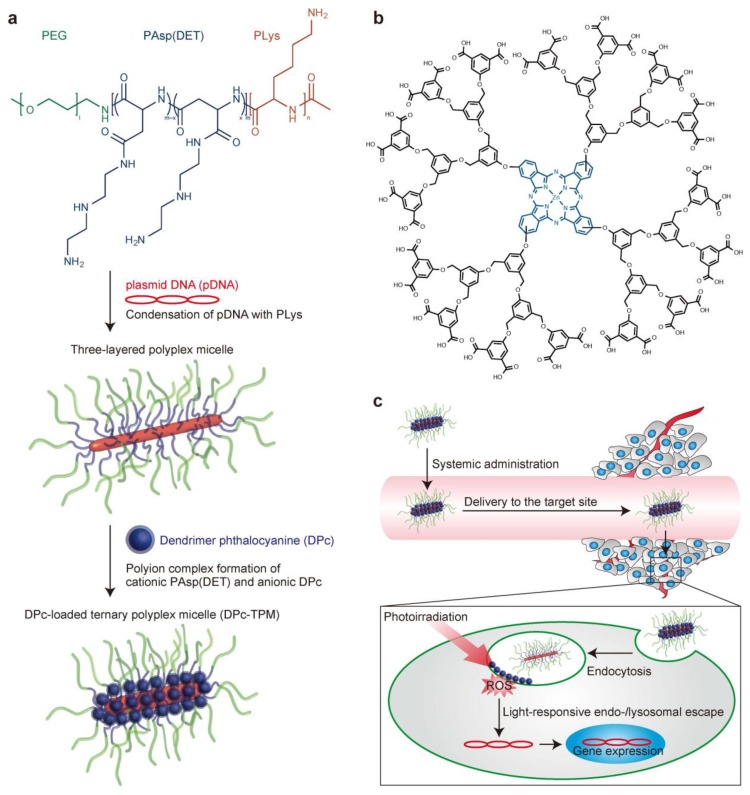
Construction of the DPc-TPM and light-responsive gene transfer. (**a**) Design of the DPc-TPM. First, a three-layered polyplex micelle is prepared by mixing PEG-PAsp(DET)-PLys triblock copolymer and pDNA; the polyplex micelle is composed of a PEG shell, an intermediate PAsp(DET) layer and a PLys/pDNA core. The DPc-TPM is constructed by adding DPc to the PAsp(DET) intermediate layer; (**b**) Chemical structure of DPc; (**c**) Scheme showing the delivery at systemic and intracellular levels. Reproduced with permission from [89].

Another category of pH-sensitive assemblies is the polyplex micelles that fabricated from DHBCs through charge neutralization of the oppositely charged polyion blocks [86,87]. The controlled swelling and disassembly of the cross-linked cores can be actuated upon cellular endocytosis. The pH-responsive polymer segments can transform from neutral state to the charged state once subjected to acidic microenvironment in endosomes, the “proton sponge’’ effect in endosomes will result in the destabilization of endosomes and promote to endosomal escape, avoiding the deactivation of biotherapeutics. As a typical application, high efficiency gene delivery carriers based on amino acid containing water-soluble block copolymers have attracted great attention in the past decade. Kataoka et al. reported an A-B-C type triblock copolymers, poly(ethylene glycol)-poly[(3-morpholinopropyl) aspartamide]-poly(l-lysine) (PEG-PMPA-PLL), consisting of three distinctive functional segments for constructing gene delivery systems [88]. PEG was used as the hydrophilic biocompatible segment, PMPA segment with a p*K*_a_ of 6.2 possessed the capacity of buffering, and PLL segment was used to condense DNA due to the much higher p*K*_a_ (~9.4). The PEG-PMPA-PLL triblock copolymers could form three-layered polyplex micelles consisting of a pDNA/PLL core, an intermediate layer of PMPA, and an outer shell of PEG. A significantly enhanced transfection activity through the buffering capacity of the PMPA segment and efficiently compacting pDNA by the PLL segment were achieved. Furthermore, a newly designed system of polyplex micelles as a multifunctional nanocarrier platform for light-induced gene transfer was reported by the same group [89]. A three-layered compartments within a single wormlike nanocarrier (DPc-TPM) was fabricated by the sequential self-assembly of plasmid DNA and photosensitizer (dendrimer phthalocyanine; DPc) with PEG-PAsp(DET)-PLys triblock copolymers through electrostatic interaction. The DNA-packaged core was covered by the photosensitizer-incorporated intermediate layer, which was encompassed by an outer shielding PEG shell (Figure 3). In the target tumor sites, DPc-TPMs were taken up by cells via endocytosis and entrapped in endo-/lysosomes. In response to the low pH environment in the endo-/lysosome, DPc was released from the DPc-TPMs owing to the protonation of the peripheral carboxyl groups and interacted with the endo-/lysosomal membrane through hydrophobic interactions. Upon following photoirradiation, DPc generated reactive oxygen species that could destabilize the endo-/lysosomal membrane, facilitating endo-/lysosomal escape of DNA and displaying >100-fold gene expression in cultured cells. 

Similarly, the hydrophobic poly(β-amino ester) (PAE) could exhibit pH-responsive hydrophobic-hydrophilic transition characteristics due to the presence of tertiary amine moieties. Micelles composed of PAE blocks were also exploited to encapsulate drugs, showing pH-dependent micellar stability [90,91,92]. For example, Shi et al. developed a self-regulated delivery system based on the controlled self-assembly of PEG-*b*-PCL and a targeting ligand c(RGDfK) modified PCL-*b*-PAE (PCL-*b*-PAE-c(RGDfK)) in water [93]. The micelles with mixed shell of hydrophilic PEG and pH-responsive hydrophobic PAE were obtained. The c(RGDfK) ligand was conjugated to the collapsed PAE domain and hidden in the shell of PEG at pH 7.4. At tumor pH, charge conversion of PAE domain occurred, and the c(RGDfK) ligands stretched out of the shell, leading to facilitated cellular internalization and drug release. These PAE containing mixed-shell micelles could rapidly and reversibly change surface properties due to the protonation/deprotonation effect, resulting in prolong circulation time in blood (pH 7.4) and enhanced cellular uptake at tumor sites (pH 6.5), and endowing them with adaptive surfaces to match the process of nanoparticles transporting in/out of tumors.

**Figure 4:**
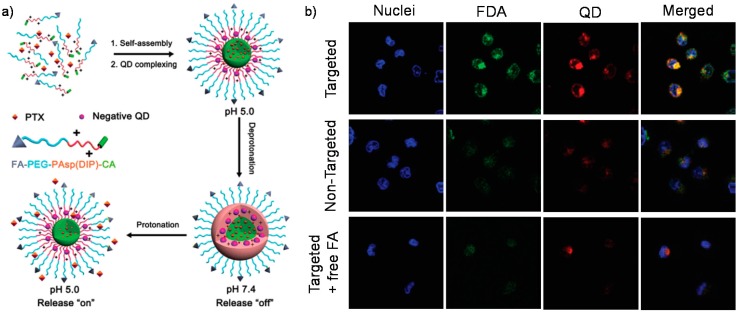
(**a**) Illustrative preparation of drug (PTX) and QDs-loaded micelles and pH-tunable drug release; (**b**) Confocal laser scanning microscopic images of Bel-7402 cells incubated with FDA and QD-loaded micelles (incubation time: 3 h; in the free FA competing assay cells were incubated in the presence of 1mg/L FA). Reproduced with permission from [95].

It should be noted that the acidic microenvironment of intracellular endosomes and lysosomes is not unique to tumor cells, normal cells can be also response to acid responsive delivery systems. Thus, the therapeutic efficacy of drug nanocarriers based on pH-responsive assemblies mainly relies on the targeting capacity to tumor cells over normal cells [94]. To achieve this purpose, Shuai et al. introduced folic acid (FA) units to functionalize the diblock copolymers *folate*-PEG-*b*-poly(*N*-(*N’*,*N’*-diisopropyl-aminoethyl)aspartamide)-cholic acid (FA-PEG-*b*-PAsp(DIP)-CA) for active tumor targeting based on the fact that folate receptor is over-expressed in many cancer cells [95]. In aqueous solution, the diblock copolymers could self-assemble into micellar structures. The interlayered PAsp(DIP) exhibited pH-responsive drug release under intracellular acidic lysosomal conditions (pH ~5.0), showing effective tumor suppression (Figure 4a). The FA-targeted advantage was confirmed by synchronously fluorescent imaging assisted by quantum dots (QDs), cells incubated with the FA-targeted micelle showed much stronger FDA and QD fluorescence in comparison with the cells incubated with the non-targeted one (Figure 4b).

## 4. Enzyme-Sensitive Assemblies

**Figure 5:**
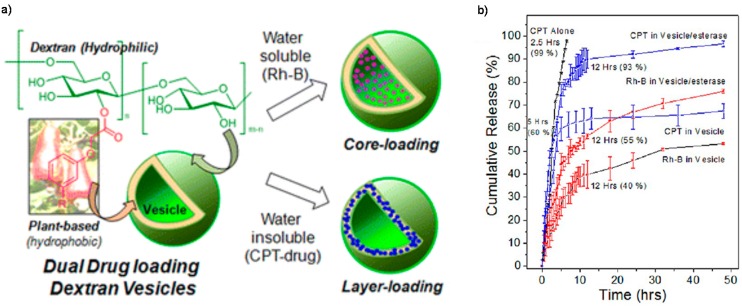
(**a**) Schematic illustration of dextran vesicular approach for capture of hydrophilic and hydrophobic drugs (or molecules); (**b**) Cumulative drug release of CPT, CPT loaded vesicles, and RhB loaded vesicles. Reproduced with permission from [100].

Another powerful tool to cleave the linkers in polymer-drug conjugates for drug release is enzyme [96,97,98,99], which have emerged to be a promising triggering motif and played a crucial role in the regulation and function of cells and tissues. Enzyme-catalyzed reactions are always highly selective and efficient toward specific substrates under mild conditions. Typically, Balasubramanian and Jayakannan et al. anchored 3-pentadecylphenol and its unsaturated counterpart cardanol to the dextran backbone via aliphatic ester linkage to develop nontoxic amphiphilic dextran derivatives, which could self-organize in water to form dual encapsulating dextran vesicles for drug loading and delivering hydrophilic as well as hydrophobic molecules into cells (Figure 5a) [100]. Esterase was then used as stimuli to break the polymer scaffold to release CPT or RhB from the vesicular scaffolds at significantly much faster rate (Figure 5b).

Drug molecules covalently linked to polymer scaffold via a peptide linker are always responsive to specific enzyme, which are always present with much higher concentrations and abnormally activity in the tumor tissue compared to that in normal tissues due to the over expression [101,102]. Thus, enzyme-catalyzed reactions are frequently used to image and diagnose the occurrence certain diseases, such as early stage tumors. The most convenient construction approach is the direct conjugation of hydrophobic drug molecules to a hydrophilic polymer chains via an enzyme cleavable linkage. For example, Duncan and Vicent et al. designed a HPMA copolymer based conjugates, PHPMA-GPLG-AGM-Dox, where PHPMA is poly(*N*-(2-hydroxypropyl) methacrylamide) that have progressed to clinical trials, GPLG is a tetrapeptide linker Gly-Phe-Leu-Gly that known to be cleaved by lysosomal thiol-dependent protease cathepsin B, the first-generation aromatase inhibitor (aminoglutethimide, AGM) is chosen as the endocrine model [103]. In the presence of lysosomal enzymes, Dox and AGM were released from the PHPMA-GPLG-AGM-Dox conjugates by the cleavage of tetrapeptide linker, showing synergetic endocrine therapy and chemotherapy effect to elicit improved antitumor activity in breast cancer. Moreover, Langer et al. synthesized dextran-peptide-methotrexate conjugates for tumor-targeted delivery of chemotherapeutics via the mediation of tumor-associated enzymes, matrix metalloproteinase II and matrix metalloproteinase IX, intending to combine the concepts of polymer-drug conjugate and peptide prodrug in their conjugates and exploit the presence of metalloproteinase in tumor vicinity to release active drug molecules [104]. The Pro-Val-Gly-Leu-Ile-Gly sequence was served as peptide linker and the charges on the dextran backbone were fully neutralized. The conjugates had satisfactory stability in serum containing conditions and remained intact in systemic circulation. In the presence of the targeted enzymes, the peptide linker was cleaved and peptidyl methotrexate was released.

**Figure 6:**
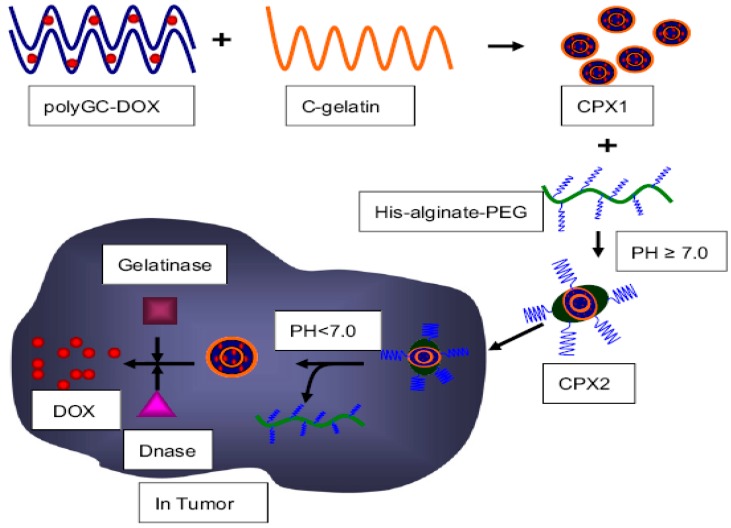
Fabrication scheme of CXP1 and CPX2, as well as working mechanism in tumor tissue. Reproduced with permission from [107].

On the other hand, site-specific delivery and triggered drug release from nanocarriers can also be achieved by employing enzyme-responsive polymers, whereas conventional polymers responsive to simple stimuli cannot easily complete all the tasks [21,105,106]. The combination of enzyme-catalyzed reactions with stimuli-responsive polymers can further expand the design diversity and future applications by endowing the polymer scaffold with enhanced triggering responsibility. For dual stimuli-driven disintegration, a pH and enzyme dually responsive tumor site-specific delivery system for Dox was reported by Zhang et al. [107]. In this work, a negatively charged polyGC-Dox complex, constructed from Dox and double stranded oligoDNA, was mixed with a positively charged cationic polymer (C-gelatin), which led to the formation of CPX1 through the electrostatic interactions. Then, a pH-sensitive polymer (His-alginate-PEG; p*K*a: ~6.9) was used to cover the surface of CPX1 to form CPX2, which was stable under normal biological milieu and could avoid undesirable accumulation in the liver. Once exposure to the acidic microenvironment (pH < 7) in tumor tissue, pH-sensitive His-alginate-PEG firstly left the CPX2 due to electrostatic repulsion. The naked CPX1 was further degraded in the presence of gelatinase and deoxyribonuclease (DNase), resulting in the effective release of encapsulated Dox (Figure 6). The synergetic effects of pH-triggered protonation and eventually enzyme-catalyzed Dox release accomplished the highly site-specific drug delivery and localized drug release within tumor tissues.

In contrast, synthetic polymers possessing enzyme-reactive moieties in the form of labile linkages along the polymer main chain or side groups can also exhibit triggered self-assembling and aggregation characteristics [108,109,110]. The enzyme-triggered hydrophilic-amphiphilic or sol-gel transition from initially water-soluble block copolymers could be achieved with highly selective and substrate-specific manner in aqueous media under mild conditions. The triggered assembly protocol endows these aggregates with excellent biocompatibility and potential applications as drug/gene nanocarriers for bioactive purpose.

**Figure 7:**
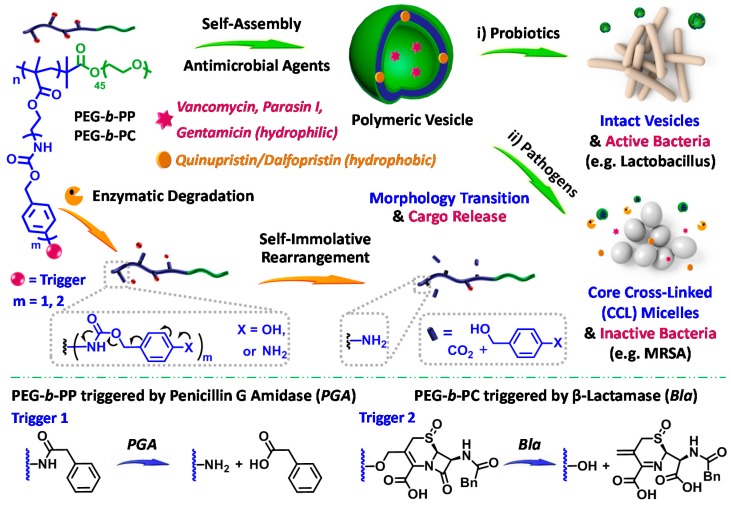
Enzyme-responsive polymeric vesicles for bacterial strain selective delivery of antibiotics. Polymeric vesicles self-assembled from PEG-*b*-PP and PEG-*b*-PC are subjected to side chain cleavage and microstructural transformation in response to penicillin G amidase (PGA), and β-lactamase (Bla), respectively. This process is accompanied with sustained release and bioactivity recovery of antimicrobial agents encapsulated within vesicles. Reproduced with permission from [111].

In addition, antimicrobial resistance poses serious public health concerns and antibiotic misuse/abuse further complicates the situation, thus, it remains a considerable challenge to optimize/improve the usage of currently available drugs. From this point, Liu et al. reported a general strategy to construct a bacterial strain-selective delivery system for antibiotics based on responsive polymeric vesicles [111]. The side chain enzymatic cleavage mechanism was introduced in the material design, and penicillin Gamidase (PGA) and β-lactamase (Bla) associated with bacterial resistance were chosen as target enzymes. PGA and Bla-responsive polymeric vesicles were self-assembled from amphiphilic diblock copolymers consisting of hydrophilic PEG block and hydrophobic block containing enzyme-cleavable self-immolative side linkages, termed as PEG-*b*-PP and PEG-*b*-PC, respectively. During vesicle formation, antimicrobial agents were loaded into either hydrophobic bilayers or aqueous interiors. Once the vesicles are subjected to enzymatic uncapping of terminal groups and subsequent self-immolative cleavage, hydrophilic poly(2-aminoethyl methacrylate) scaffolds were formed (Figure 7). By involving side chain degradation triggered by drug-resistant enzymes and final nanostructures with comparable chemical compositions, controlled degradation kinetics, predictable payload release profiles, and targeted antibiotic delivery were successfully achieved.

## 5. Redox-Sensitive Assemblies

The significant redox potential difference between the extracellular (oxidation) and intracellular (reduction) microenvironments endow the drug delivery systems a new mechanism. Disulfide bonds, the most frequently studied reduction-responsive groups, favor the oxidizing environment in the extracellular microenvironment and can maintain the structure and functions [112,113,114]. However, the intracellular reducing condition due to the presence of over-expressed glutathione (GSH; 2–10 fold higher than that in normal tissues) in cancer cells will cleave the disulfide bonds to release free thiol groups as well as loaded drugs [115,116,117]. Several reduction-responsive polymeric nanocarriers concerning thiol-triggered intracellular drug release and imaging have already been reported. For example, Boyer and Davis et al. reported a type of amphiphilic copolymers consisting of hydrophilic POEGMA and hydrophobic block containing both methanethiosulfonate (MTS) and pentafluorophenyl ester (activated ester) pendant functionalities [118]. The following thiol/MTS exchange reaction was used to modify the hydrophobic segments with different functional groups. The copolymers were self-assembled in water yielding nanoparticles with activated esters moieties in the core. A pH-sensitive ketal bearing difunctional amino cross-linker was then used to cross-link the nanoparticles to generate core cross-linked (CCL) micelles. Drug encapsulation and release was modeled using hydrophobic (Nile Red) and hydrophilic (thiol-modified fluorescein isothiocyanate) dye molecules, demonstrating the possibility of selective release of single dye or the simultaneous release of both dyes depending on the experimental stimuli (sulfhydryl compound or pH). These disintegrable CCL micelles exhibited several integrated advantages, such as excellent biocompatibility, superior drug loading, high extracellular stability, and triggered intracellular drug release.

Although encapsulation of small-molecule drugs into copolymer assemblies has been extensively used for preparing polymeric nanocarriers, the inevitable low drug loading efficiency tends to be a major obstacle to limit the therapy efficiency. To address this challenge, a dimeric drug conjugate bearing a trigger responsive domain was designed by Cheng et al. [119] and used as the core constructing units of the nanocarriers. Specifically, camptothecin (CPT), a well-known anticancer agent, are stably conjugated to the 2,6-bis(hydroxymethyl)-aniline via carbonate linkages that are subject to triggered bond cleavage and subsequent drug release by a reducing reagent. The amine group of the aniline is protected by a disulfide bond bearing short chain. Upon triggering, cleavage of the disulfide bond would result in decomposition of the drug dimer, releasing CPT in its authentic form. This distinct structure makes the drug molecules structurally less rigid, not only preventing long-range order of drug packing and formation of large drug aggregates, but also largely enhancing the drug loading.

**Figure 8:**
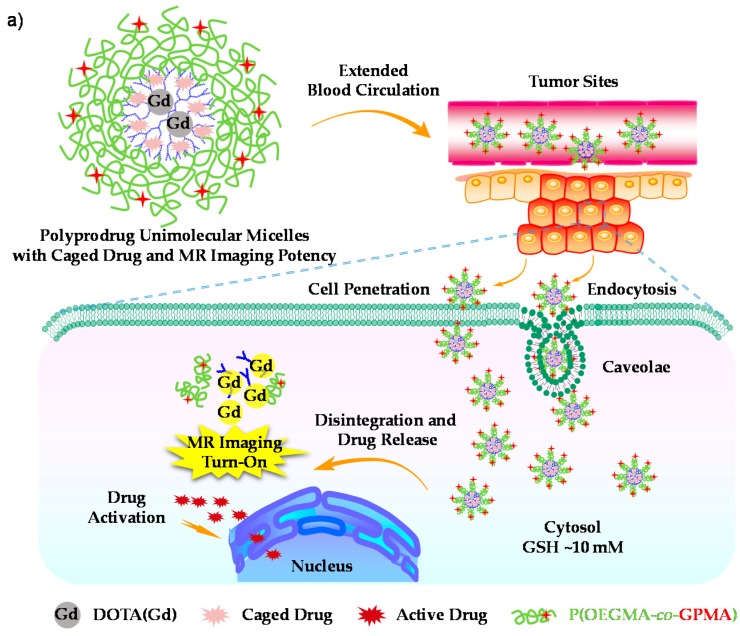

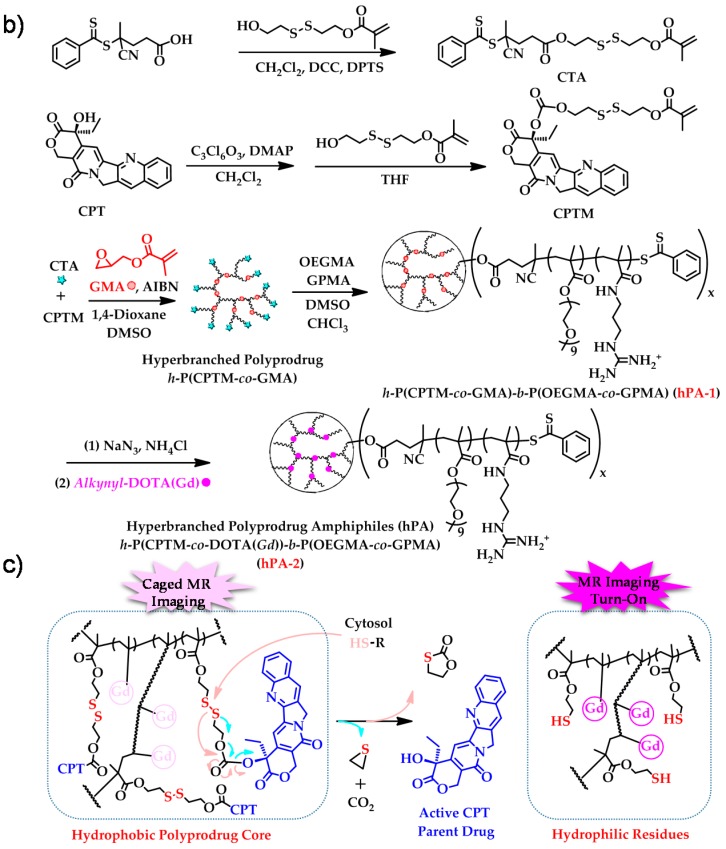
(**a**) Schematic illustration of polyprodrug unimolecular micelles with hyperbranched cores conjugated with DOTA(Gd) and reductive milieu-cleavable camptothecin prodrugs and hydrophilic coronas functionalized with guanidine residues; (**b**) Reaction schemes employed for the synthesis of hyperbranched polyprodrug amphiphiles, *h*-P(CPTM-*co*-DOTA(Gd))-*b*-P(OEGMA-*co*-GPMA) (hPA-2); (**c**) Proposed mechanism of reductively activated CPT drug release in cytosol milieu from hyperbranched polyprodrug cores and concurrent hydrophobic-hydrophilic transition of the local milieu surrounding the Gd complex. Reproduced with permission from [121].

Furthermore, Liu et al. described the synthesis, hierarchical self-assembly, and relevant biological functions of amphiphilic block copolymers, PEG-*b*-PCPTM, where PEG is poly(ethylene glycol) and PCPTM is polymerized block of reduction-cleavable CPT prodrug monomer [120]. These diblock copolymers (>50 wt % CPT loading content), termed as *polyprodrug amphiphiles*, can self-assemble into four types. Among them, the unprecedented staggered lamellae assemblies outperform the other three nanostructure types. The controlled hierarchical organization of polyprodrug amphiphiles and shape-tunable biological performance opens up new horizons for exploring next-generation block copolymers based drug delivery systems with improved efficacy. This work represents the typical example of polyprodrug amphiphiles which are amenable to the fabrication of multiple hierarchical nanostructures for the investigation of shape-tunable biological performance. Based on this remarkable excogitation, a new class of theranostic nanoparticles constructed from hyperbranched polyprodrug amphiphiles consisting of hyperbranched cores conjugated with reduction-activatable CPT prodrugs and MRI contrast agent (Gd complex), and hydrophilic coronas functionalized with guanidine residues was designed by the same group [121]. The hyperbranched cores could avoid the potential interactions between CPT and blood components and served as the embedding matrix for T1-type MR contrast agents to weaken MR background signals. Upon cellular internalization, the synergistic turn-on of therapeutic potency and enhanced diagnostic imaging in response to tumor milieu were achieved (Figure 8). In addition, guanidine decorated hPAs showed extended blood circulation and excellent tumor cell penetration potency. Such superior synergistic imaging/chemotherapy capability and enhanced tumor uptake endow the hyperbranched chain topology to be a validly candidate to design novel theranostic polyprodrug platform. As a newly emerging field, further efforts might consider the types of polyprodrug amphiphiles and in vivo stimuli-triggered degradation efficiency.

Reactive oxygen species (ROS), such as hydrogen peroxide (H_2_O_2_), hydroxyl radicals (•OH), superoxide radicals (O_2_^−^), hypochlorite (OCl^−^), and singlet oxygen (^1^O_2_), play a really important role in both healthy and diseased biological processes [122]. A high local concentration of ROS will damage cellular DNA, protein, and lipid molecules, leading to a variety of diseases, including cancers and Parkinson’s and Alzheimer’s diseases, which are termed as “oxidative stresses” [42,43].

**Figure 9:**
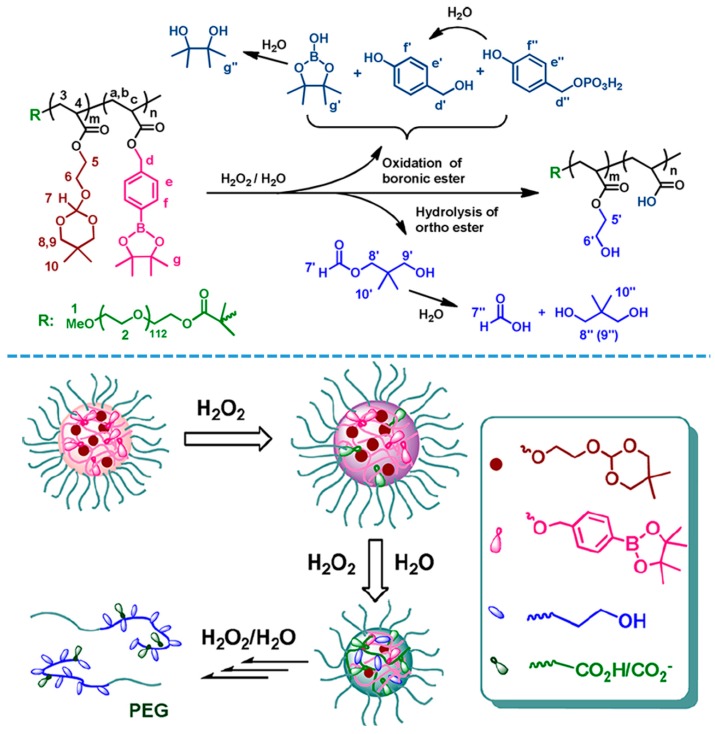
The reaction mechanism illustration of phenylboronic ester oxidation and ortho ester hydrolysis in the presence of H_2_O_2_/H_2_O. Reproduced with permission from [123].

A variety of block copolymer-based oxidation-responsive systems have been developed and utilized for different purpose, take H_2_O_2_ for example, Li et al. reported a pH and H_2_O_2_ dual-responsive amphiphilic block copolymer composing of a PEG block and a hydrophobic segment containing different amounts of pH-sensitive ortho ester and H_2_O_2_-sensitive phenylboronic ester pendent groups [123]. The copolymers could self-assemble into micellar structures in phosphate buffer, the phenylboronic ester oxidation was relied on the H_2_O_2_ concentration and as well accelerated the following up ortho ester hydrolysis (Figure 9). Moreover, the micelles were extremely sensitive to the biorelevant concentration of H_2_O_2_ (50 μM) at pH 7.4, suggesting great promise for inflammation specific drug delivery. Similarly, another kind of phenylboronic ester functionalized amphiphilic block copolymers was reported by Liu et al. [124]. The presence of H_2_O_2_ could trigger the transformation of amphiphilic charge-generation polymers from initially uncharged state into a cationic polyelectrolyte on demand by taking advantage of the chemo-selective cleavage of phenylboronic-carbamate protecting moieties. The charge-generation process was then coupled with the induced aggregation of Au nanoparticles to design colorimetric probes.

**Figure 10:**
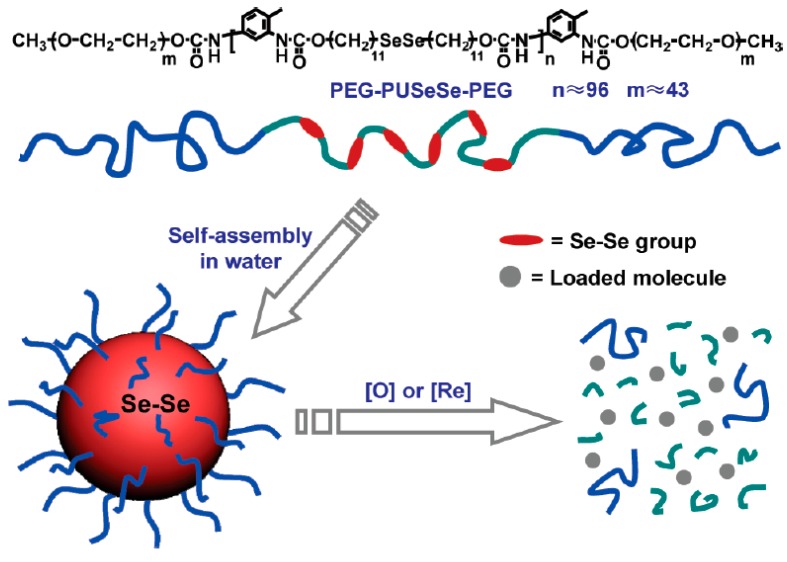
Structure of PEG-PUSeSe-PEG and schematic of the redox responsive disassembly of PEG-PUSeSe-PEG micelles. Reproduced with permission from [126].

Another example of the redox-responsive polymeric drug delivery system was selenium-containing block copolymer assemblies, since diselenide (Se–Se) bonds (bond energy: 172 KJ/mol) can be either oxidized to seleninic acid in the presence of oxidants or reduced to selenol in a reducing environment [125]. For example, Zhang and Xu reported an ABA-type main-chain diselenide-containing amphiphilic triblock copolymers, poly(ethylene glycol)-poly[urethane(SeSe)]-poly(ethylene glycol) (PEG-PUSeSe-PEG), which exhibited good solubility in common solvents and self-assembled in aqueous solution to form micelles [126]. The redox-induced disassembly of the RhB loaded-micelles could be realized by the addition of oxidant (H_2_O_2_) or reductant (GSH) (Figure 10), leading to the rapid RhB release within 5 h. This work explores a new direction for the preparation of block copolymer capable of backbone cleavage ability and burst release of payload in cellular environment.

## 6. Light-Sensitive Assemblies

Light, with variable wavelength and intensity, is a cheap and easily manipulated clean resource and can be used in a spatiotemporal precision manner. Cargo-loaded ultraviolet (UV) light-responsive polymeric micelles and vesicles have gradually received much attention due to their potential applications in the diagnosis and treatment, such as quick and localized drug release and high-efficiency on-demand disease treatment compared with other formulations [127,128,129,130]. The already reported UV-responsive nanocarriers are mainly concerning two different mechanisms: hydrophobicity-hydrophilicity transition and photo-triggered cleavage or degradation induced drug release. The former light responsive nanocarriers always include a hydrophilic corona, such as PEO, and a photochromic group bearing hydrophobic core; while, the latter are generally fabricated from self-immolative polymers, which can respond to multiple stimuli and then a spontaneous head-to-tail cascade depolymerization reaction occurs and produces amplified response outputs.

**Figure 11:**
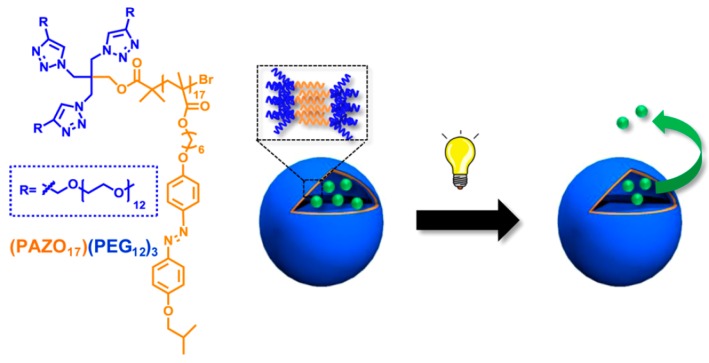
Chemical structure of the AB_3_ type miktoarm copolymers and schematic of the light responsive disassembly of vesicles and subsequent drug release. Reproduced with permission from [131].

Amphiphilic block copolymers containing one hydrophilic block and another photo-sensitive group bearing hydrophobic block can self-assemble into chromophore incorporated micellar or vesicular based nanocarriers, whose hydrophobicity-hydrophilicity balance can be conveniently adjusted by photo irradiation. Because of the rapid, reversible, and high quantum yield photo-induced structure transformation, azobenzene (AZO; photoisomerization process) [131,132] and spiropyran (SP; reversible ring-opening/closing reaction) [133,134] have been widely studied for designing light-responsive block copolymers and corresponding assemblies. Upon irradiation with 340–380 nm UV light, the nonpolar planar *trans*-azobenzene groups were transformed to the more polar nonplanar *cis*-forms, which significantly increased the hydrophilicity of the original hydrophobic blocks. This process can return back under irradiation at 420–490 nm or place in the darkness. Therefore, the assemblies could be dissociated with UV light and reassembled using visible light in a reversible manner. For example, Oriol and coworkers reported an AB_3_ type miktoarm copolymers possessing one polymethacrylate (PAZO) arm with 4-isobutyloxyazobenzene side chain and three PEG arms (PEG), denoted as (PAZO)(PEG)_3_, which could form stable polymeric vesicles in water [131]. After encapsulating both hydrophilic and hydrophobic fluorescent probes, the external stimulus to trigger the release of probes was performed by irradiation with UV light (*λ* = 350–400 nm), causing the disruption of the vesicles and cargos release (Figure 11).

**Figure 12:**
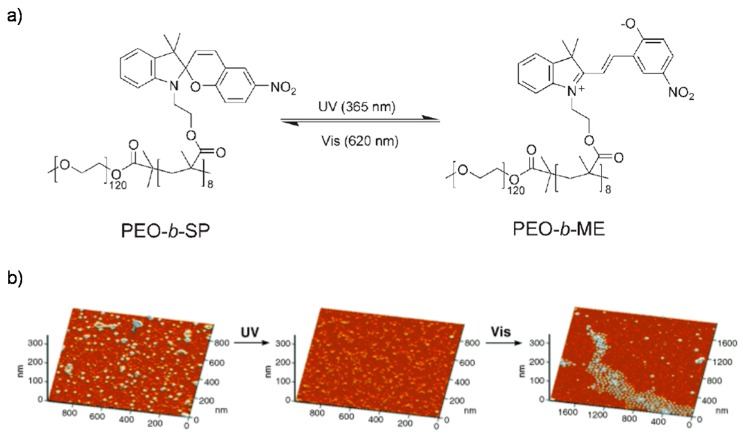
(**a**) Light-induced isomerization between PEO-*b*-SP and PEO-*b*-ME by alternating irradiation of UV and visible light; (**b**) AFM height images of PEO-*b*-SP micelles before and after exposure under 365-nm UV as well as followed by 620-nm visible-light for 30 min, respectively. Reproduced with permission from [133].

On the other hand, the SP group can reversibly isomerize from a hydrophobic closed-ring form (colorless; SP) to a hydrophilic charged opened-ring merocyanine form (colored; ME) under UV (365 nm) and visible light irradiation (620 nm). Compared to AZO moieties, the difference in polarity between hydrophobic SP and hydrophilic ME units is greater, hence, the SP-incorporated polymers with reversible behavior have been widely studied for various applications such as light-actuated nanovalves and nanoswitches. For example, PEO block SP-containing methacrylate monomers were reported by Matyjaszewski group [133], which termed as PEO-*b*-PSP (Figure 12a). In aqueous solution, the block copolymer could self-assemble into spherical polymeric micelle with hydrophobic SP-containing inner core and hydrophilic PEO outer shell. These micelles could be completely disassembled upon UV irradiation and reversibly regenerated by visible light (Figure 12b). When a hydrophobic dye was encapsulated within the hydrophobic core, its release could be induced by UV light. However, a portion of the released dyes could be re-encapsulated when the micelles were irradiated with visible light, resulting from the recovery of micellar morphology.

**Figure 13:**
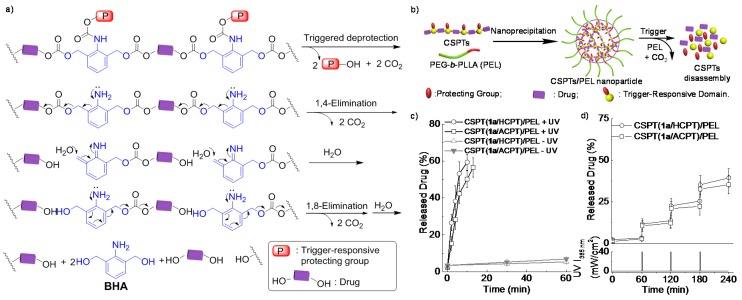
(**a**) Degradation of 2,6-bis(hydroxymethyl)aniline (1) carbonate units in a CSPT by 1,4- and 1,8-elimination reactions (two repeating units shown in the scheme); (**b**) Preparation of CSPT/PEL nanoparticles by nanoprecipitation, disassembly of the nanoparticles in response to trigger-induced CSPT degradation, and drug release from the nanoparticles; (**c**) Release of drugs from nanoparticles with continuous UV irradiation (+UV) or without UV (−UV) irradiation; (**d**) Pulsatile release of drugs from nanoparticles in response to periodic UV irradiation for 1 min every 60 min. Reproduced with permission from [140].

Furthermore, *ο*-nitrobenzyl (NB) derivatives, for example, have been incorporated into amphiphilic block copolymers and proved to be an efficient method for constructing photo-triggered cleavage or degradation nanocarriers [135,136,137,138,139]. The NB moieties can be placed in the main chain or side chain of polymers. The former may lead to entire disruption of assembly structures into small molecules or low molecular weight fragments after light irradiation, resulting in a burst and complete release of the cargos. The latter, exhibiting similar hydrophobicity-hydrophilicity transition, may lead to partial or entire disruption of assembly structure and sequential cargo release after light irradiation. Although NB is now widely employed in the design of trigger-responsive drug delivery system, especially in self-immolative system, incorporating a responsive linker that allows active release of the conjugated drug remains synthetically challenging. Cheng et al. used multiple drug and trigger responsive domain (TRD) molecules as monomers to construct a self-immolative TRD/drug type of condensation polymer, serving as a new polymeric therapeutic (PT) platform with precise control over both drug loading and release [140]. The resulting platform had specific repeating units and molecular structure. 2,6-Bis(hydroxymethyl)aniline was used to condense with a diol drug to form a PT platform with UV light responsive carbonate bonds. Once the UV-sensitive *ο*-nitrobenzyloxy-l-carbonyl protecting group was removed, the PT could undergo a 1,4-elimination followed by a 1,8-elimination, leading to chain shattering and the release of the constituent drug molecules (Figure 13a). Moreover, by co-precipitating chain-shattering polymeric therapeutics (CSPTs) with poly(ethylene glycol)-*block*-poly(l-lactide) (PEL) in water, the CSPTs/PEL nanoparticles (*d* < 150 nm) with extremely high drug loading (>48 wt %) and loading efficiency (>92%) were obtained (Figure 13b). These nanoparticles showed excellent responsiveness to light-induced drug release. Without UV irradiation, the proportion of drug released from the nanoparticles in phosphate buffered saline (PBS) solution was negligible. However, when the nanoparticles were irradiated for 10 min, 59% of the drug was released (Figure 13c). Pulsatile release of drug from nanoparticles was also observed with periodic UV irradiation (Figure 13d). As an emerging research filed, the responsive linker incorporated self-immolative system and the nanostructure fabrication need further investigation, including more potent types, various chain topologies, and other stimuli-cleavable linkages. 

**Figure 14:**
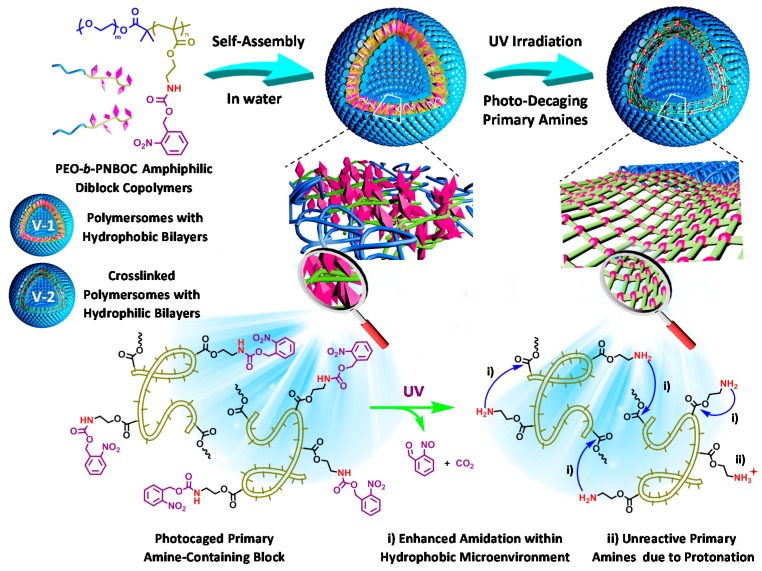
Design of block copolymer vesicles exhibiting concurrent phototriggered “traceless” crosslinking and vesicle membrane permeabilization. PEO-*b*-PNBOC amphiphilic block copolymers self-assemble into polymersomes with the hydrophobic bilayer containing carbamate-caged primary amine moieties. UV irradiation triggers decaging reactions and the release of primary amine functionalities, prominent amidation reaction then occurs because of a suppressed amine p*K*_a_ within the hydrophobic vesicle membrane, resulting in vesicle crosslinking instead of vesicle-to-unimer disassembly. (1) Enhanced amidation within the hydrophobic microenvironment. (2) Unreactive primary amines because of protonation. Reproduced with permission from [141].

Recently, Liu et al. chanced upon the solution of a long-standing dilemma concerning the synchronized stability and permeability of vesicles (polymersomes) by employing a light-regulated “traceless” crosslinking strategy [141]. Briefly, amphiphilic block copolymers, PEO-*b*-PNBOC, with the hydrophobic PNBOC block containing photolabile carbamate-caged primary amine moieties were designed. Upon self-assembling into vesicles, UV-triggered self-immolative decaging could release primary amine groups and result in hydrophobicity-to-hydrophilicity transition of bilayer. At the same time, the free amine groups took part in the subsequent vesicle crosslinking by amidation reactions, leading to enhanced vesicle stability instead of vesicle-to-unimer transition due to the generation of hydrophilic polymer chains (Figure 14). This feature had been further successfully used to achieve light-regulated co-release of both hydrophobic and hydrophilic substances and light-switchable biocatalysis of enzyme entrapped vesicle nanoreactors. However, the hydrophobicity-to-hydrophilicity transition and subsequent light-regulated “traceless” crosslinking process is irreversible and the bilayer cross-linking is unidirectional transition; moreover, the photo-triggered release of small molecule reactive intermediates (2-nitrosobenzaldehyde) might further incur cytotoxicity issues. To overcome such issue, poly(ethylene oxide)-*b*-PSPA (PEO-*b*-PSPA) diblock copolymers, where SPA is SP-based monomer containing a unique carbamate linkage, were prepared by the same group [142]. Upon self-assembling into polymersomes, SP moieties within vesicle bilayers undergo reversible phototriggered isomerization between hydrophobic spiropyran (SP, λ > 450 nm) and zwitterionic merocyanine (MC, λ < 420 nm) states. For both SP and MC polymersomes, their microstructures are stabilized by multiple cooperative noncovalent interactions including hydrophobic, hydrogen bonding, π–π stacking, and paired electrostatic (zwitterionic) interactions. The experiment results confirmed that the carbamate-incurred hydrogen bonding interactions in PEO-*b*-PSPA were crucial for polymersome stabilization in the zwitterionic MC state, and the reversible phototriggered SP-to-MC polymersome transition was accompanied by membrane polarity and permeability switching from being nonimpermeable to selectively permeable toward noncharged, charged, and zwitterionic small molecule species below critical molar masses. The photoswitchable spatiotemporal release of 4’,6-diamidino-2-phenylindole (DAPI, cell nucleistaining dye) within living HeLa cells was further demonstrated.

## 7. Multi-Sensitive Assemblies

Although great progress has been made in the field of stimuli-responsive drug carriers, more intricate designs and innovative strategies need to be developed to adapt to the more complex situations. For example, most of the investigated stimuli-responsive block copolymer delivery systems are only responsive to a single stimulus, which are not capable of integrating more complicated delivery and release needs. Thus, engineering sequential or simultaneous multi-stimuli responsive delivery systems will facilitate more intricate drug delivery and controlled release processes and imaging ability [143,144,145]. An earlier example was reported by Jiang et al. that a kind of thermo and pH dual-responsive nanoparticles, assembled from P(NIPAM-*co*-AA)-*b*-PCL, with appropriate response to pathological environment and high payload of anticancer drug (paclitaxel; PTX) was fabricated [146]. The nanoparticles aggregated at body temperature under a slightly acidic pH (~6.9), and a faster drug release was found at elevated temperature and low pH values. This property led to selective accumulation of these nanoparticles and subsequent drug release in tumor tissues, which is advantageous in targeted anticancer drug delivery. 

Recently, a library of water-dispersible hyperbranched self-immolative polymers (hSIPs) with structurally and functionally diversity were fabricated by utilizing one-pot AB_2_ polycondensation methodology and sequential postfunctionalization [145]. The spontaneous radial depolymerization of hSIPs upon stimuli-triggered single cleavage of the capping moiety allowed the construction of myriad functions including visible light actuated intracellular release of conjugated chemotherapeutic drugs in a targeted and spatiotemporally controlled manner, intracellular delivery and cytoplasmic reductive milieu-mediated pDNA release, mitochondria-targeted fluorescent imaging and sensing of H_2_O_2_ with a high detection limit, and colorimetric H_2_O_2_ assay by integrating triggered dispersion of gold nanoparticle aggregates with enzyme-mediated cycle amplification. The modular design, facile synthesis, multifunctional construction, water-dispersibility, high intrinsic chemical amplification, and potency of integrating with external cycle amplification modules of the reported hSIPs platform opens a new avenue to fabricate novel stimuli responsive materials with high selectivity, sensitivity, and specificity, a prerequisite for functions in complex biological media. Moreover, the fabrication of self-immolative polymersomes via self-assembly from amphiphilic block copolymers consisting of a hydrophobic multi-triggered self-immolative block and a hydrophilic poly(*N*,*N*-dimethylacrylamide) (PDMA) block was also reported [144]. The self-immolative block was caged with perylen-3-yl, 2-nitrobenzyl, or disulfide moieties, which are responsive to visible light, UV light, or reductive milieu, respectively. Upon removal of caging moiety by appropriate stimulus, the block copolymer were depolymerized and subsequent triggered drug corelease and controllable access toward protons, oxygen, and enzymatic substrates were achieved.

## 8. Conclusions and Perspectives

With the rapid development of polymer synthetic and nano techniques, stimuli-responsive block copolymers and corresponding assemblies were created one after another and used for targeted delivery of drugs and imaging/contrast agents to tumor sites and realized controlled/triggered release. In this review, recently emerged tumor and intracellular microenvironment-responsive block copolymer assemblies with diverse functions, structures, and self-assembling morphologies have been discussed. Typical tumor microenvironments, such as weak acidic pH, abnormal temperature gradients, a variety of specifically over-expressed enzymes, and redox species are available to be utilized to construct responsive block copolymer based integrated platform, allowing for triggered payload release and enhanced imaging sensitivity. Although great progress in this field has been achieved, not all of the therapeutic effects are performed as well as expected. The materials that respond to physiological relevant concentrations within micromolar or nanomolar regime are still lag behind requirement.

The common feature of the above mentioned examples is that all the selective and controlled modulation processes were based on the stimuli-responsiveness of polymeric building blocks, and the accumulation in tumor sites is assisted by either passive or active targeting. However, the nonnegligible low percent (sometimes only 0.7% [147]) of injected dose accumulated in the tumor sites is indeed one of the most challenging issue and has not been solved yet, which is also problematic for stimuli-responsive assemblies. Worm-like micelles with long circulation time, high accumulation, and deep penetration capabilities, are a potential solution. It is necessary and valuable for developing novel stimuli-responsive worm-like assemblies for theranostic applications [148]. Moreover, both systemic and cellular barriers will affect the efficient delivery of conventional nanocarriers to tumor sites and subsequent endocytosis more or less. How to conveniently guide the functional nanocarriers into cancer cells more facilely is an urgent problem need to be solved. Considering the development tendency, further efforts might consider the introduction of cell-penetrating motif to the nanocarrier design. Nowadays, because of their superior interactions with cell membranes, positively charged cell-penetrating peptides (CPPs; with a large number of arginine residues) and its analogue as well as guanidine-decorated copolymers are performed as an efficient medium to facilitate intracellular delivery of various cargos, including small molecules and macromolecules, regardless of the scaffold. Some well-known CPPs either adopt inherent helical structures or form helices in the cell membranes and confirm that the trans-membrane helix is essential for their membrane interactions and promoting their cellular uptake [149,150,151]. Shortly before, Cheng et al. reported that positively charged helical poly(arginine) mimics showed superior cell membrane permeability up to two orders of magnitude higher than that of TAT peptides, as well as excellent DNA and siRNA delivery efficiencies in various mammalian cells [152].

However, the artificial noncharged polymer mimics exhibiting cell-penetrating properties are rarely emerged. Poly(phenyl isocyanide) (PPI) is a class of interesting synthetic polymers because it can maintain stable helical conformation in solution and in solid state. Yashima et al. reported that PPI possessing a stable rigid rod-helical conformation with a long persistence length [153]. These helical polymers are proven to be ideal building blocks for self-assembly of 2D and 3D smectic arrangements on a substrate, in solution and solid state. Several works concerning the different substituted helical PPI have been reported by us [154,155,156,157], and obtained a series of advanced morphology and performance. Therefore, we envision that introducing noncharged hydrophilic helical PPI chains that has a structural similarity to CPPs in the micelle shell may also facilitate intracellular delivery of cargos and create an artificial synthetic noncharged polymer-based delivery system. Moreover, more potent prodrug types and other stimuli-cleavable linkages to enrich the chance for biomedical applications are other key challenges.
